# General practitioners' and practice nurses' views and experience of managing depression in coronary heart disease: a qualitative interview study

**DOI:** 10.1186/1471-2296-13-1

**Published:** 2012-01-05

**Authors:** Elizabeth A Barley, Paul Walters, André Tylee, Joanna Murray

**Affiliations:** 1 Section of Primary Care Mental Health, Health Services and Population Research Department, PO Box 28, Institute of Psychiatry, King's College London, De Crespigny Park, London, SE5 8AF, UK

## Abstract

**Background:**

Depression is common in coronary heart disease (CHD). Affected patients have an increased incidence of coronary symptoms and death. Little is known about how best to manage primary care patients with both CHD and depression. This study is part of the UPBEAT-UK programme of research and was designed to understand general practitioners' (GPs) and practice nurses' (PNs) views and experience of managing depression in CHD.

**Methods:**

Individual in-depth interviews with 10 GPs and 12 PNs in South East London. Data were analysed using constant comparison.

**Results:**

GPs and PNs had similar views. Distress following diagnosis or a cardiac event was considered to resolve spontaneously; if it endured or became severe it was treated as depression. GPs and PNs felt that psychosocial problems contributed to depression in patients with CHD. However, uncertainty was expressed as to their perceived role and responsibility in addressing these. In this respect, depression in patients with CHD was considered similar to depression in other patients and no coherent management approach specific for depression in CHD was identified. An individualised approach was favoured, but clinicians were unsure how to achieve this in the face of conflicting patient preferences and the treatment options they considered available.

**Conclusions:**

GPs and PNs view depression in CHD similarly to depression uncomplicated by physical illness. However, uncertainty exists as to how best to manage depression associated psychosocial issues. Personalised interventions are needed which account for individual need and which enable and encourage clinicians and patients to make use of existing resources to address the psychosocial factors which contribute to depression.

## Background

Coronary Heart Disease (CHD) can cause distressing symptoms and functional limitation. The prevalence of depression in CHD patients has been estimated at 20%[1]. Depression increases the incidence of coronary symptoms and death in CHD patients independent of other factors [1]. It may also exacerbate the perceived severity of symptoms and increase service use [2].

Concurrent physical illness reduces the recognition of depression by GPs [3]; accordingly, in the UK, GPs are now remunerated for screening CHD patients for depression [4]. Antidepressants and CBT have been found to improve mood in CHD, although physical health outcomes have not improved [5,6]. A recent trial of collaborative care, an enhanced depression care intervention which provides depression severity related treatment guidance, found improvement in both depression and control of medical disease at 1 year post intervention in patients with heart disease and/or diabetes [7].

Patients with depression and or CHD are managed mostly in primary care. However, although there has been much work concerning general practitioners' (GPs) and practice nurses' (PNs) management of depression in primary care [8]; little is known concerning how they manage patients with both conditions. A recent qualitative study aimed to determine barriers to managing depression in people with CHD or diabetes [9]. Interviews and a focus group with healthcare professionals, service users and carers indicated that depression was often normalised in the presence of long term conditions (LTCs) and that performance managed environments in primary care militated against shared understandings of depression. However, the views of PNs were underrepresented in this study given that, as a group, they have the most regular contact with such patients.

Similar tensions between delivering care to meet quality targets and fulfilling the patients' agenda were found in an interview study of GPs' and PNs' perceptions of the management of patients with multimorbidity in general [10]. Issues specific to CHD were not considered in this study. The current study was conducted as part of a NIHR funded research programme: UPBEAT-UK [11]. It explores GPs' and PNs' views and experience of managing depression in patients with CHD and its findings will inform the development and implementation of strategies within the programme to help primary care staff manage such patients effectively.

## Methods

### Sampling

The sampling frame was 31 GP practices participating in the UPBEAT-UK cohort study of patients with depression and CHD. They were from 4 ethnically and socially diverse boroughs in South East London (Lambeth, Lewisham, Southwark and Croydon).

A purposive, maximum variation approach was used based on ethnicity, age, practice setting (inner city *versus *suburban) and type (single handed *versus *group). Male and female GPs were recruited; no male PNs were identified. After several interviews, it was noted that participants often mentioned their involvement in the UPBEAT-UK cohort study and we became concerned that this involvement might have increased awareness of depression in CHD. From then on, only clinicians whose practices were enrolled in UPBEAT-UK but who were not personally involved were interviewed. Snowballing was also used to identify participants independent of UPBEAT-UK. Level of participant involvement in UPBEAT is indicated in Table 1. Recruitment stopped when saturation of themes was reached; that is, no new themes or information relating to the identified themes emerged.

**Table 1:** Participant characteristics

	GPs	PNs
**Age (years)**		
**Range**	24 - 63 yrs	33 - 59 years
**Mean**	47.6 years	43.3 years
**Median**	48 years	42 years
**Gender**		
**Female**	3	12
**Male**	7	0
**Ethnicity**		
**White British**	4	10
**African/Afro-Caribbean**	3	0
**Asian**	3	2
**Practice setting**		
**Mainly deprived**	4	3
**Mixed**	5	9
**Mainly affluent**	1	0
**Practice type**		
**Single handed**	1	1
**Group**	9	11
**UPBEAT-UK involvement**		
**Involved**	8	5
**Practice recently recruited/participant not yet aware**	2	4
**None**	0	3

### Data Collection

EB conducted all the interviews using a guide based on literature review [8]. Broad topics were: understandings of depression and detecting and managing CHD depression. Prompts were used to elicit opinions on topics identified in the literature search, such as the use of screening tools and differences between 'distress' and 'depression'. Prompts were revised iteratively, for instance, early participants introduced the problems of 'erectile dysfunction' and 'housebound patients'; these were explored with later participants. In order to ground opinions in practice, participants were asked to recall specific patients with CHD and depression. Interviews were recorded and transcribed verbatim by EB. Participants gave written informed consent.

### Analysis

Interviews and analyses were performed concurrently using principles of constant comparison [12] and thematic analysis [13]. Three researchers (EB, JM and PW) coded independently the first interview and agreed descriptive codes. EB and JM independently applied these and, where appropriate, new codes to the following 4 transcripts when consistency in coding was achieved. Descriptive codes were collated into themes and a preliminary explanatory framework devised. This was used as the basis for coding and for informing future interviews. Data for each theme were gathered and coded by EB using computer software (NVIVO 8 [14]). The robustness of themes was tested by examining differences and similarities between coded data. A sample of coding was agreed between two researchers (EB and JM). Theoretical memos [15] containing ideas and impressions from interviews and transcripts were produced and used to inform coding discussions.

## Results

### Participants

We interviewed 10 GPs, 11 PNs and one clinical pharmacist from 12 practices. The pharmacist's role was similar to the PNs', but she was more involved in medication management. Since during the analysis her views were not found to differ from that of PNs, we treated her data as PN data. We would have recruited more clinical pharmacists, but we are not aware of any others working within practices enrolled in UPBEAT-UK. This pharmacist had an important role in managing CHD patients within her practice so we felt that her views were important and that our data would not be complete without them. Participant characteristics are shown in Table 1. All contacted agreed to participate.

Diverse views were expressed, but, on the whole, divergence was not found to be related to participant group i.e. the GPs, the PNs, the community pharmacist, those involved in UPBEAT or those less or not involved. For most themes a majority view independent of participant profession was identified, that is GPs and PNs had similar views. GP and PN data are therefore combined except where differences were found; these are reported. The themes identified are described, with quotations identified by profession (GP = general practitioner, PN = practice nurse) and the interview order (GP1-10; PN1-11; P1 clinical pharmacist).

### Theme: Recognising depression

#### Distress versus Depression

The participants reported difficulty distinguishing in general between 'distress' and depression needing treatment. They were aware that many patients with or without CHD experienced difficult social circumstances. It was therefore 'understandable' that they felt low.

"When they come to the clinics there is some level of depression. Whether it's due to their disease, it's difficult to say. I think there is a lot of other things in this area that cause that." (P1)

Similarly, in CHD most participants felt that distress following diagnosis or a cardiac event was 'natural'.

"I guess if someone was to come in with recently being diagnosed with CHD and came in a particularly low mood. Initially, again, you might just put it down to the fact that they've been diagnosed with quite significant illness, so you may not call it depression as such." (GP7)

The potential for sudden death and the feeling of vulnerability this produces were highlighted as particularly distressing.

"....something with your heart, everyone knows that the heart is such an important organ, don't they? And they, and everyone thinks 'well, if it stops, that's it'." (GP8)

For most people with CHD, it was thought that distress resolves spontaneously, although no time period was specified. For many, the level of impact of CHD on life was also important.

"Part of it is their disability due to their disease, erm, but not their disease *per se*. 'Cause if they're functioning OK, I don't seem to find that there's an issue. Whereas, if they are actually, you know, 'I can't walk far, I'm breathless' all of that, then yes there is." (P1)

Distress and depression in CHD therefore appeared to be conceptualised similarly to that in other illnesses and on a continuum of chronicity and/or severity. Only distress that becomes chronic and /or severe was considered to require management.

"You know, if it's someone who's just feeling crap for a day, you know, that doesn't warrant it [*management*], but feeling crap for a long time does........something about the, you know, severity and the chronicity is important." (GP2)

"Some hospitals put patients on antidepressant, I've noticed, quite quickly, sometimes even before they've been discharged, which I sometimes worry about because obviously the event is all a bit new then and, and if they are tearful or really distressed it's sort of erm understandable that they are in a way." (GP8)

#### Depression Screening

Most participants regularly used the two screening questions stipulated in the quality and outcomes framework (QOF) of the UK general practice contract [4]. Several used the Patient Health Questionnaire 9 (PHQ9) [16] or the Hospital Anxiety and Depression Scale (HADS) [17] following a positive response to screening. In some practices these were not available to PNs.

Most felt that depression in CHD is under-diagnosed. This may be because some patients consider it inappropriate to mention mood during a consultation about CHD, or because they fear mental health-related stigma or causing discomfort. However, screening instruments helped some clinicians initiate a conversation about mood in a non-threatening manner.

"we're saying 'it's not actually our fault - we've been told to do this by big brother. So actually, it's OK to talk about it'. So it's been very helpful from that point of view. It's kind of taken the stigma off asking and responding." (GP3)

For several participants, these instruments raised awareness of depression in CHD.

"Now that I've actually been asking the questions, I've picked up people that, actually, looking back, I've known it for years and I haven't done anything about it." (GP3)

Reservations were also voiced; these tended to relate to depression screening in general not just in CHD. Several participants, especially PNs, said that they avoided using them due to a fear of uncovering unmanageable problems.

"I'm bad at asking, in some ways I think, like lots of nurses, you don't want to open up something that you then, then can't deal with afterwards" (PN11)

One Asian participant (P1) felt that South Asian patients conceptualise depression in somatic terms and that these instruments would not detect this. In contrast, another participant felt that the instruments detected somatic symptoms which could be confused with depression.

"Some of them *[patients] *misinterpret it *[PHQ-9]*, because, I mean some of them might/when they're older, they find they don't sleep quite so much and they expect to still sleep 12 hours a night. And you do find that a lot of them, do sort of say they have problems sleeping and there could be other factors that are influencing that more than because they are depressed." (PN3)

#### Clinical judgement

Most participants also valued their clinical judgement. They used this to decide when to ask just the QOF questions or to give a more detailed questionnaire, or to supplement the information obtained by such measures. Most agreed that if they felt the QOF questions were not providing a 'true picture' they would use their clinical judgement. A range of depression indicators was described including crying, frequent attending, sleep disturbance, reduced activity, tiredness, loss of appetite or non-attendance at appointments. Several participants felt they could recognise depression from the patient's demeanour. For some, this involved intuition; others noted signs such as a head down stance, lethargic manner, fixed gaze or lack of eye contact. Several, however, noted that a 'jolly demeanour' may mask depression, which was an argument for active screening.

"Some of them surprise me - you think 'oh yes, they're fine.......and you get them to fill in this form and you think 'oh!'" (PN3)

No strategies for assessing depression specifically in patients with CHD were identified.

### Theme: GP and PN perceptions of why some CHD patients become depressed

Possible physiological links between depression and CHD were raised by only one GP.

"if one's stressed and one's stress hormones go up, one's platelets get more sticky and the endothelium gets more sticky and all that sort of happens. And also if one's got cardiovascular disease that may influence peoples' neurotransmitters." (GP3)

A number of factors commonly associated with CHD such as loss of a valued role (e.g. loss of employment), inability to fulfil responsibilities due to disability and erectile dysfunction were considered to lead to depression. Erectile dysfunction was considered especially important with most participants agreeing that men are reluctant to report this. Despite this observation and the availability of specialist clinics, most GPs and PNs did not ask about this routinely.

"No I don't, no. Again, I wish, I mean, I should do *(ask about erectile dysfunction) *because that's something that we can offer them as well for that." (PN11)

Nurses may also be embarrassed to introduce this topic; one PN suggested that being older helped.

"it's probably easier for me because I'm a lot older and maybe they're not so embarrassed. So if I can bring it up, then it can be a lot more sort of open." (PN9)

Other CHD related factors thought to contribute to depression were feeling responsible for their illness and having to make unwanted lifestyle changes to prevent CHD progression.

"I think many people, as perhaps part of their CHD depression, feel guilty about it: 'yes I did inflict it." (GP1)

"You know the sort of modification in their lifestyle and things can be really, really difficult. If it's somebody that's been smoking for example and is trying to give up smoking and life feels like it's not worth living cause they can't smoke ....." (P1)

Several participants considered that depression may lead to heart disease as depressed individuals are more likely to lead an unhealthy life.

Social problems such as financial and housing difficulties were thought to be related to depression and were considered common among CHD patients. Isolation was mentioned by almost all participants.

"Social, loneliness - very important, loneliness, loss of employment, isolation, the home environment. Sometime they need their home to be adapted to their, to their physical and medical needs at the time and they will not have it. But most importantly is loneliness." (GP5)

Other predisposing factors for depression cited were not necessarily considered related to CHD. For instance some participants mentioned lack of resilience, poor coping skills and 'premorbid personality' (GP1).

"I think that while most of us maybe will cope with stress and anxiety, there is a core population that if they are tipped to a very severe extent will dip into a depressive phase. Presumably, that's inherited, it's constitution, it's related to our chemical make up." (GP9)

Lack of education was thought problematic, although one PN felt the educated were more at risk due to the stress arising from a greater awareness of potential complications. Some patients were thought to hold negative attitudes to their CHD which could be disabling independent of disease severity.

"It's their perception that they're an invalid and quite often they're not an invalid, maybe they could go back to work." (PN2)

Alcohol or drug use and a past history of depression were also mentioned,

### Theme: depression management

All participants felt that treating depression would lead to improvements in self management of CHD, which suggests that they are motivated to address this issue.

"Cause sooner or later, someone with depression is going to say 'why bother about my statins, my cholesterol, my diet - who cares? Why do the exercise? Smoking - well actually I find it quite comfortable? I'm not interested in will I get a lung cancer in 10 years time or not, I can't see ahead for 10 years'. Whereas if somebody feels really optimistic, positive then you're gonna be thinking 'yes, I'm doing all this to ensure my own better future." (GP1)

"if you've treated their depression, their outlook on life might be better as a whole, so therefore they want to remain well, so they're taking their medication, not just their medication, their exercise their food, whatever, smoking..... so it ..... all goes hand in hand." (PN10)

Several GPs and PNs stressed the importance of patient choice in increasing adherence to management programmes.

"Of course they've got to come to it themselves, because if you're going to offer any sort of therapy or treatment, it's a complete waste of time if they haven't got to actually saying 'well, yes I want it'. (GP9)

Individual GPs and PNs raised and discussed a variety of management options for depression; sometimes these were related to depression comorbid with CHD but more often participants did not differentiate between this and depression in general.

#### Antidepressants

Several GPs and PNs felt that antidepressants were useful in 'lifting' a patient's mood to the point that they would be able to return to normal functioning. However, GPs had treated only a few CHD patients in this way. The majority only prescribed antidepressants in CHD when other options had been exhausted, in severe depression, suicidal intent, if mood was deteriorating or if a patient had responded well previously.

"It isn't always drug treatment, it's about going through the rehab programme, getting the confidence to go out and do things, starting driving again, having sex - all thse sorts of things sometimes are therapeutic.......so very often medication is not always needed". (GP9)

Hesitation in prescribing was related to a perceived reluctance in patients to accept antidepressant treatment due to fear of stigma or a general dislike of medication.

"We discuss with the patients. You know, depends where the patients stand, yes and then minority of the cases go on antidepressant tablet/treatments, you know, not everyone wants treatment." (GP10)

This was not necessarily associated with the patient's CHD, although patient dislike of medication was considered increased when they were taking multiple drugs as most CHD patients are.

"she has so many tablets anyway and she's always wanting to stop this and stop that and 'can I just cut this down?' and 'can I just miss out my asprin for a couple of days?'.....To add another tablet, an antidepressant, into the mix would just probably be the thing that tipped her over the edge."(PN5)

Only one GP was concerned about drug interactions.

Several of the PNs were not prescribers, but, among those who were, there was reluctance to prescribe antidepressants due to a lack of confidence in managing depression.

"I have been prescribing a few years now, but I do find I tend to stick to things I'm happy with and that I deal with a lot, which is CHD, diabetes, women's health, travel health family planning........but because it *[mental health] *is something that I don't deal with a lot, I'm not happy to prescribe. So I do tend to ask advice before I would prescribe." (PN3)

"I am a nurse prescriber, but I wouldn't feel comfortable or sort of competent enough to do that" (PN9)

#### ii) Talking therapy

Mostly the generic term 'counselling' was used, although a few participants referred to 'CBT' or 'psychotherapy'. Counselling was widely favoured by both GPs and PNs to help patients come to terms with their condition, to increase confidence in self management or to aid in venting feelings.

"I think counselling would definitely be number one on the list. I mean a lot of the time, you just ask the question 'would you like someone to talk to?' And then a lot of the time they will say 'yes'. So rather than ....medicalising it too much, you could maybe try simple steps like counselling services, support groups, helplines. And that might just be enough ...... to improve their mood." (GP7)

"sometimes just seeing a counsellor and getting things off their chest for a few sessions will help." (PN9)

Three GPs said they, or another GP within the practice, provided counselling such as 'mini' CBT, problem solving therapy or '10 minute CBT'. Otherwise, a counsellor or psychologist (or both) was available in most of the practices. Despite this, a lack of availability of counselling was commonly raised; all but two PNs said that waiting lists were too long. They complained that this meant they were unable to follow treatment guidelines which promote the use of talking therapy.

"Because our waiting lists are so long, so although all the guidelines....say counselling treatment ect, we haven't got primary care counselling really". (P1)

Only one GP mentioned the Improving Access to Psychological Therapies (IAPT) programme: they felt that primary care practitioners were not yet fully aware of it. This may be expected as, at the time of the study, this was a relatively new service in the area. Computerised CBT was considered by a few participants to be unsuitable for elderly CHD patients who may not be computer literate. Reluctance to undertake therapy was also observed due to perceived stigma or denial.

Some PNs reported that they were not authorised to make counselling referrals; they did not complain about this. This may further reflect uncertainty among nurses in managing depression which was summed up by one PN:

"I could do *[make a referral to a counsellor]*. On the whole, I prefer to do it through the GP, just in case the GP doesn't agree that they need it." (PN9)

#### iii) Informal counselling

This involved providing education about CHD and assurance that distress is normal. GPs tended to refer patients to a counsellor if this took too long. Most PNs agreed that this is part of their role; several had CHD patients who would come in for 'a chat'. Some would schedule extra consultations for this, despite being unsure how useful it was. They did not know what else to do however.

"At the moment, I dunno what to do with this group of people, so I see them more regularly because I feel that they need contact with somebody, but I dunno if that's the best thing to do...."(PN5)

#### iv) Exercise

Some participants recommended exercise to improve mood.

"I explain to them about serotonin levels - how if you do exercise you can produce more and it makes/it's a happy hormone and all the rest of it." (PN6)

The social aspect of 'exercise on referral' schemes and 'seated exercise classes' was considered beneficial.

#### v) involvement of other agencies

One GP reported having made a psychiatry referral when she did not know how to progress, but was not helped.

"I actually referred him up to psychiatry, because I felt, he was actually very vulnerable and very at risk of suicide. I felt, 'cause he was very isolated, he lost his job, he's relatively young. But the psychiatrists wouldn't see him, they just bounced it back and said 'you know, oh you're doing a good job with your medication, nothing more we can do'." (GP9)

Generally, it was felt that the Community Mental Health Team (CMHT) was for complex cases and so they would not deal with depression in CHD patients or depression generally.

"then we have CMHT and other services - erm not hugely accessible for this kind of this level of mental health problems." (GP2)

Cardiac rehabilitation was considered helpful but poorly attended by some patients, such as working people and Asian women reluctant to attend a mixed class. Only one GP had liaised with cardiac rehabilitation in the management of a depressed patient.

"Rehab is some mythical thing in primary care I think! It just takes place in the hospital and that's that." (PN5)

Lack of communication was also reported between primary care staff and district/community nurses (DNs) who manage housebound CHD patients. Some PNs did not know what DNs did, although they suspected that they do not address psychological needs due a heavy workload which prioritises physical health.

"It's quite sad really, but we don't have a lot to do with our district nurses in this practice. I think if they've got concerns they speak to the duty doctor. But we as a whole, we don't sort of link in with each other. I don't know them and they don't know us.." (PN10)

"I just don't know whether the district nurses go into it *[mood] *very much, 'cause they are usually so busy. They, they sometimes just tick the boxes like, you know, the blood pressure's been done and what it is and and 'yes, they are on asprin'." (PN9)

One PN made home visits to housebound CHD patients in order to gain QOF points. However, a PN at a different practice believed these patients were excluded from QOF registers and so they did not receive any depression screening or management.

"those patients probably get exempt from their registers because they are housebound.........'cause I think that if you prove that you've written or invited them three times and they haven't come in then you can exempt them." (PN8)

One GP also noted that talking therapy is not available for housebound patients.

"one of the, the quite striking things is that there's almost no access to talking therapies for people who are housebound. There are, you know, people who are frail, elderly or with things like heart disease who may be rather more likely to be housebound, but, you know, counsellors and psychologists are pretty much, you know, practice or clinic based and don't go and visit people at home." (GP2)

A perception of a relationship between depression and social problems, irrespective of the presence of CHD, led a few clinicians to direct their patients to community facilities, such as church coffee mornings and local libraries. However, they found it difficult to identify such resources.

"it's really, it's knowing what, what is available because I am sure there's lots of things out there, but it's just really knowing....." (PN11)

Furthermore, some participants either did not see resolution of social problems as their responsibility or felt powerless to help. This seemed to be especially the case for PNs, perhaps because they have more time to talk to patients about their problems.

"'cause there's nothing I can do for them. ....'cause actually what can I do? I can help your physical things, but actually if you've got issues with your extended family at home, I can't do anything about that." P1

"There might be something about the grandchild or something or the children and there's not a lot you can do about that" (PN2)

In contrast, one practice had a social prescribing service where a professional directed patients with identified social problems to appropriate agencies. Staff at this practice reported many patients with complex social needs; one GP stressed her pleasure in working with such patients. This attitude was promoted and a flexible attitude to time management was adopted.

"I, personally, I **really **like our population and find them interesting......Their problems are quite complex. It's rare for them to come in with a single problem..........and if they come in with their 3 problems, and actually one problem's going to take up the whole of the consultation, more often than not they will get more than what they would have got if the/it's unusual for someone to say 'no, that's it, you've, you've had your, your time'. 'Cause we generally don't work like that." (GP8)

## Discussion

The GPs and PNs in this study identified factors associated with CHD such as feelings of responsibility for having caused their illness, unwanted lifestyle changes, loss of employment, inability to fulfil responsibilities due to disability and erectile dysfunction that they felt may be associated with depression, However, these may not be CHD specific and may be important in other long term conditions (LTCs). Other predisposing factors for depression which are unrelated to LTCs were also raised such as social problems, individual differences and coping skills. On the whole, the GPs and PNs did not differentiate between depression in patients with CHD and depression in other patients; it was thought that individuals may 'naturally' become distressed following a diagnosis of CHD or a cardiac event, but only when distress becomes severe and enduring is it seen as depression requiring treatment. This view of depression as a natural reaction to life events has been found in studies of the management of depression uncomplicated by physical illness [18-20].

The GPs and PNs in this study felt that depression is under-diagnosed in CHD. However, their opinions concerning the use of screening instruments varied. A study [21] of GPs' use of depression screening questionnaires showed that, although doctors used them, they preferred to rely on their 'practical wisdom and clinical judgement'. Some of our participants shared these views and many PNs did not even have access to questionnaires such as the PHQ9 [16] or the HADS [17]. Some GPs however reported positive applications, and most used their clinical skills as a supplement to screening data or to help to decide whether to use a more detailed questionnaire.

When managing depression uncomplicated by CHD, GPs have been found to favour 'watchful waiting' over antidepressants [22]. Similarly, in our study, antidepressants were not the GPs' first choice. Reluctance in CHD patients to accept antidepressants was reported; this was felt to be either due to fear of mental health-related stigma or to negative attitudes towards medication in general which may be amplified in patients who require multiple medications for co-morbidity. Talking therapies were favoured, but few participants differentiated between approaches such as CBT or supportive counselling which may lead to less appropriate referrals. Some patients were observed to reject talking therapy due to fear of stigma. However, the main barrier was a lack of availability, as reported previously [18]. In the UK, the Government's Improving Access to Psychological Therapies programme [23] is addressing this, but at the time of this study this was a relatively new innovation and availability was not consistent across the boroughs in which the participants worked. Informal counselling, such as reassurance and education, was also discussed; most GPs were unwilling or unable to give much time to this. Some PNs reported that they did have time, but doubted its usefulness.

The GPs and PNs reported liaising rarely with other professionals when managing patients with CHD and depression despite guidance [24] promoting this. Greater use by health care professionals of services, such as social clubs and advice agencies, which promote well being has also been encouraged [24]. Knowledge of such services varied widely between our participants. This may relate to our finding of variation in attitudes to managing social problems. Nurses were especially concerned about social problems, perhaps because they reported spending more time providing informal counselling and so had greater opportunity to probe these issues. Our recent meta-synthesis [8] found also that management of depression uncomplicated by physical illness is perceived by primary care staff in the UK as particularly complex when patients present with social problems; both GPs and PNs in the included studies were aware of the relationship between social and mood problems but they were unsure of its exact nature and of their role in managing it. The participants in the current study are especially likely to encounter social problems among their CHD patients as heart disease is more common in people from lower social economic backgrounds. Previous research [19] has identified 'therapeutic nihilism' where clinicians feel helpless in the face of the complex social problems which impact on health. This was seen in several of our participants. However, one practice actively sought to address social difficulties by providing 'social prescribing'; there may be scope to develop this for depressed CHD patients.

No clear management strategy specific for patients with CHD and co-morbid depression was identified; the treatment issues and management options raised appeared similar to those in depressed patients without physical comorbidity. Collaborative care, where nurses and doctors work together to deliver evidence based treatment, has been shown to be beneficial for depression [25] and, recently, a trial conducted in USA found it to improve both depression and disease control in patients with CHD and/or diabetes [7]. However, in this study some nurses did not consider managing mental health to be their role. These PNs reported a lack of training, interest or time. Negative past experience of mental health training has been found to be associated with nurses' current negative attitudes towards managing mental health [26]. Since primary care patients with CHD commonly receive most of their care through nurse-led clinics, our findings suggest that the development of interventions for depression in these patients should include sensitive consideration of nurses' views.

### Strengths and limitations of the study

Some of our participants may have been sensitised to the link between depression and CHD by having been recruited into the UPBEAT-UK study [11]. However, given this, findings of uncertainty among clinicians in the understanding and management of this condition appear particularly important. Diverse views were expressed, but reducing complex data into themes may result in decontexualisation of speakers' words. We therefore employed a rigorous iterative, multidisciplinary approach to our analysis in order to ensure that our summaries are an accurate representation.

This study was confined to South East London; however we recruited participants from contrasting areas (inner city, suburban, deprived, affluent) with a range of experience and characteristics in order to elicit a range of experiences. Also, many of the current findings are supported by previous research conducted in other contexts and so are likely to be broadly representative. Finally, this study was conducted prior to the introduction in the UK of guidelines for the management of depression in adults with a chronic physical health problem [27]; these may impact on attitudes and practice.

## Conclusions

In this study, GPs and PNs identified CHD related factors that they felt may be associated with depression, but also other predisposing factors such as social problems which can occur in any depressed population. The importance of social factors may be increased in people with CHD as they are especially likely to come from lower socioeconomic backgrounds, but this may also be true for other LTCs. Our participants, in common with those of studies of depression uncomplicated with physical comorbidity, expressed uncertainty as to how to address depression associated with psychosocial problems. In the face of perceived individual differences in the causes of depression in CHD, an individualised treatment approach was favoured but clinicians were unsure how to achieve this in the face of conflicting patient preferences and the treatment options they considered available. This suggests that flexible interventions are needed which enable and encourage clinicians and patients to make use of existing resources, such as social clubs and advice agencies, to address the psychosocial and other factors which contribute to depression.

## Competing interests

The authors declare that they have no competing interests.

## Authors' contributions

EB conducted the interviews and the analysis and wrote the first draft of the manuscript. PW and AT conceived the study, assisted in the analysis and interpretation of data and revised the article. JM conceived the study, conducted the analysis and revised the article. All authors read and approved the final manuscript.

## Authors' Information

EB is a practitioner health psychologist, registered general nurse, researcher and systematic review module leader for the MSc in mental health service and population research at the Institute of Psychiatry.

PW is a research fellow and consultant psychiatrist.

AT is a GP, professor of primary care mental health and Academic Director of the Mood, Anxiety and Personality Clinical Academic Group at Kings' Health Partners, King's College London.

JM is a senior lecturer in social research specialising in qualitative studies in mental health.

## Pre-publication history

The pre-publication history for this paper can be accessed here:

http://www.biomedcentral.com/1471-2296/13/1/prepub
